# Evidence for Schizophrenia-Specific Pathophysiology of Nicotine Dependence

**DOI:** 10.3389/fpsyt.2022.804055

**Published:** 2022-01-27

**Authors:** Heather Burrell Ward, Adam Beermann, Uzma Nawaz, Mark A. Halko, Amy C. Janes, Lauren V. Moran, Roscoe O. Brady

**Affiliations:** ^1^Beth Israel Deaconess Medical Center, Boston, MA, United States; ^2^Harvard Medical School, Boston, MA, United States; ^3^McLean Hospital, Belmont, MA, United States

**Keywords:** schizophrenia, nicotine dependence, resting-state functional MRI, default mode (DMN) subnetwork, psychosis, tobacco

## Abstract

Tobacco use is the top preventable cause of early mortality in schizophrenia. Over 60% of people with schizophrenia smoke, three times the general prevalence. The biological basis of this increased risk is not understood, and existing interventions do not target schizophrenia-specific pathology. We therefore used a connectome-wide analysis to identify schizophrenia-specific circuits of nicotine addiction. We reanalyzed data from two studies: In Cohort 1, 35 smokers (18 schizophrenia, 17 control) underwent resting-state fMRI and clinical characterization. A multivariate pattern analysis of whole-connectome data was used to identify the strongest links between cigarette use and functional connectivity. In Cohort 2, 12 schizophrenia participants and 12 controls were enrolled in a randomized, controlled crossover study of nicotine patch with resting-state fMRI. We correlated change in network functional connectivity with nicotine dose. In Cohort 1, the strongest (*p* < 0.001) correlate between connectivity and cigarette use was driven by individual variation in default mode network (DMN) topography. In individuals with greater daily cigarette consumption, we observed a pathological expansion of the DMN territory into the identified parieto-occipital region, while in individuals with lower daily cigarette consumption, this region was external to the DMN. This effect was entirely driven by schizophrenia participants. Given the relationship between DMN topography and nicotine use we observed in Cohort 1, we sought to directly test the impact of nicotine on this network using an independent second cohort. In Cohort 2, nicotine reduced DMN connectivity in a dose-dependent manner (*R* = −0.50; 95% CI −0.75 to −0.12, *p* < 0.05). In the placebo condition, schizophrenia subjects had hyperconnectivity compared to controls (*p* < 0.05). Nicotine administration normalized DMN hyperconnectivity in schizophrenia. We here provide direct evidence that the biological basis of nicotine dependence is different in schizophrenia and in non-schizophrenia populations. Our results suggest the high prevalence of nicotine use in schizophrenia may be an attempt to correct a network deficit known to interfere with cognition.

## Introduction

Cigarette smoking is the leading cause of preventable death in the United States (1). In schizophrenia, the prevalence of smoking is over 60% (2), three-times higher than the general population (3). Tobacco use is the top preventable cause of early mortality in schizophrenia due to its associated cardiovascular disease, lung cancer, and respiratory illness (4). However, a biological explanation for the prevalence of smoking in schizophrenia has remained elusive.

There are several hypotheses for the increased prevalence of smoking in schizophrenia. One is that individuals with schizophrenia have a vulnerability to smoking due to biological overlap between schizophrenia-related pathology and processes involved in nicotine dependence (5, 6). Neuroimaging has provided partial support for this hypothesis. There have been abnormalities identified in the insula, dACC, and striatum in both schizophrenia and in nicotine dependent neurotypical individuals (7–9).

An alternative hypothesis is that nicotine use in schizophrenia is a method of “self-medicating” to correct schizophrenia pathology (10). Nicotine can enhance cognitive processes in smokers, non-smokers, and in those with schizophrenia (11–13). One proposed explanation is that nicotine use in schizophrenia is motivated by a need to correct the cognitive deficits of this disorder. This theory remains speculative because of a lack of evidence directly linking nicotine consumption to the biological processes underlying its cognitive benefit. To support a causal relationship between cognition and tobacco use, a common biological substrate needs to be (a) linked to cognition, (b) impaired in schizophrenia, and (c) acted upon by nicotine.

We sought to identify brain network pathology that could uniquely explain the prevalence of nicotine dependence among individuals with schizophrenia. The challenge is that there is a paucity of large imaging datasets in psychotic disorders that also includes detailed information about nicotine use. Similarly, studies of nicotine administration have largely focused on brain regions defined a priori or limited to regions responsive to the tasks employed (14). Critically, cross-sectional studies linking connectivity to smoking require validation of those same circuits e.g., with nicotine administration. These study constraints therefore require more selective datasets which have been intended to examine these circuits.

We utilized existing resting-state fMRI data from two independent cohorts (14–16) using an entirely *data-driven* approach. We sought to identify brain regions where functional connectivity correlates with nicotine use. This approach consists of two steps: in the first, multivariate distance matrix regression (MDMR) is used to identify brain regions where global connectivity correlates with nicotine use, then the identified region is used as a seed to determine the spatial pattern of how connectivity to this region varies with nicotine use (17). This spatial pattern of connectivity corresponded to specific brain networks. Based on these findings, we then tested nicotine's causal influence on these defined networks in a second, independent cohort of individuals. In this cohort, individuals with schizophrenia and controls received an acute dose of nicotine in a randomized, placebo-controlled crossover design (14).

We observed that severity of nicotine use in schizophrenia is strongly linked to individual variation in topography (18) of the default mode network (DMN) and this relationship is schizophrenia-specific i.e., not observed in neurotypical control smokers. Having observed an association between cigarette consumption and the DMN, we hypothesized that nicotine has a direct impact on that network. After considering which network features could be (1) modulated by nicotine and (2) experimentally measured, we hypothesized that nicotine affects within-network functional connectivity. We therefore sought to test the ability of nicotine to acutely modulate network connectivity in an independent cohort. In this cohort, we determined that nicotine can modulate connectivity in the DMN, thereby reversing schizophrenia-specific abnormalities and providing causal evidence for a biological basis for increased nicotine use in schizophrenia.

## Materials and Methods

### Participants

Cohort 1: In this cohort, we sought to identify brain circuits associated with nicotine dependence in schizophrenia and control individuals using a data-driven approach. Eighteen nicotine-dependent individuals with schizophrenia (*n* = 15) or schizoaffective disorder (*n* = 3) were recruited from McLean Hospital. Seventeen control nicotine-dependent participants were recruited from the community. Participants were 18–55 years old and provided informed consent approved by the McLean Hospital IRB. Participants were matched on gender and nicotine dependence severity (as measured by Fagerstrom Test for Nicotine Dependence [FTND]). To be included, participants reported smoking >10 cigarettes per day for at least 6 months, FTND of at least 4, and expired air carbon monoxide (CO) level of 10 ppm or greater. Individuals were excluded if they reported current substance abuse within the past 6 months, as defined by SCID-IV. All subjects were required to have a negative urine toxicology and pregnancy tests, and no recent alcohol use as measured by a breathalyzer.

Cohort 2: In Cohort 2, we sought to determine if nicotine causally affects the brain circuits we identified in Cohort 1 as associated with nicotine dependence. Twelve participants with schizophrenia and 12 control participants, aged 18–55 years old, right-handed were recruited from the community. Written, informed consent for a protocol approved by the Massachusetts General Hospital and McLean Hospital Institutional Review Boards was obtained from each participant. Control participants were excluded if they reported a history of a first-degree relative with a psychotic disorder. All participants were required to have negative urine toxicology screens at all study visits. Individuals with a history of neurological disorders or head injury with neurological sequelae were excluded. Expired CO was used to confirm smoking status (CO <5 ppm for non-nicotine-dependent, *n* = 16; CO > 10 ppm for nicotine-dependent, *n* = 8).

In both cohorts, diagnosis of schizophrenia or schizoaffective disorder and absence of Axis I diagnoses in controls was confirmed by Structured Clinical Interview for DSM-IV (SCID-IV) (19). Participants were not undergoing treatment for nicotine dependence.

Demographic, smoking, and clinical characteristics for each cohort are presented in Tables 1, 2. In Cohort 1, participants with schizophrenia were significantly older than controls (*p* < 0.01). Although groups were matched on FTND, nicotine-dependent individuals with schizophrenia had significantly greater lifetime cigarette use (*p* < 0.05). In Cohort 2, there were more current nicotine-dependent individuals in the schizophrenia group (6/12, 50%) than the control group (2/12, 17%), although this was not statistically significant (*p* = 0.08).

**Table 1:** Cohort 1: Demographics and clinical data.

	**Control** **(*****n*** **= 17)**		**Schizophrenia** **(*****n*** **= 18)**		***t***	***df***	***p***
	**Mean**	**SD**	**Mean**	**SD**			
Age	30.8	5.6	38.6	9.7	−2.9	27	0.007^*^
Gender (F/M)	8/9		9/9		χ2 = 0.030	1	0.86
Education (years)	14.4	2.4	12.9	2.4	1.9	33	0.06
Cigarettes per day	13.4	3.8	16.3	7.9	−1.3	25	0.19
FTND	5.4	1.4	5.7	1.1	−0.85	31	0.40
Lifetime cigarette use (pack years)	9.1	5.5	15.1	10.3	−2.2	26	0.04^*^
BPRS total score			48	13.5			
Chlorpromazine equivalents (mg/day)			539	504			

*FTND, fagerstrom test for nicotine dependence; BPRS, brief psychiatric rating scale*.

* *Statistically significant*.

**Table 2:** Cohort 2: Demographics and clinical data.

	**Control** **(*****n*** **= 12)**		**Schizophrenia (*****n*** **= 12)**		***t***	***df***	***p***
	**Mean**	**SD**	**Mean**	**SD**			
Age	38.8	10.7	36.5	7.9	0.61	20	0.55
Gender (F/M)	6/6		4/8		χ2 = 0.69	1	0.41
Education (years)	15.1	2.2	14.0	2.2	1.25	22	0.22
Current nicotine dependence	2 (16.7%)		6 (50.0%)		χ2 = 3	1	0.08
Cigarettes per day	14	5.7	12	7.4	0.47	2.3	0.68
FTND	5	1.4	5	1.7	0	2.1	1
Lifetime cigarette use (pack years)	6.9	6.9	10.2	9.9	−0.54	2.6	0.63
Nicotine dose (mg)	14	6.0	16.3	6.2	−0.94	22	0.36
BPRS total score			37.5	11.2			
Chlorpromazine equivalents (mg/day)			406	316			

*FTND, fagerstrom test for nicotine dependence; BPRS, brief psychiatric rating scale*.

* *Statistically significant*.

### Clinical Assessments

Smoking behavior was assessed, including cigarettes smoked per day. Severity of nicotine dependence was measured using the FTND. Symptom severity was assessed in participants with schizophrenia using the Scale for Assessment of Negative Symptoms (SANS) and Brief Psychiatric Rating Scale (BPRS). Craving was assessed using the Tiffany Questionnaire of Smoking Urges (QSU), and withdrawal was measured using the Minnesota Nicotine Withdrawal Scale (MNWS).

### Study Design

Cohort 1: In this observational study, participants underwent resting-state fMRI and clinical characterization including daily tobacco use (15, 16).

Cohort 2: A randomized, double-blind, placebo-controlled, crossover design was utilized in which each participant was administered either transdermal nicotine or identical placebo patch on two separate study sessions performed at least 7 days apart (14). The order of drug was counterbalanced within each group (schizophrenia, control). Participants received different doses of nicotine depending on smoking status (nicotine-dependent individuals were dosed based on packs per day to avoid withdrawal, range 14–28mg). Non-nicotine-dependent individuals initially received 14 mg but due to adverse events of nausea and vomiting (participants excluded from analysis, as in 13), the dose for non-nicotine-dependent individuals was reduced to 7 mg in five participants. Three hours after patch application, participants underwent resting-state fMRI. This time interval was chosen to coincide with peak serum nicotine concentrations (20) and to avoid peak withdrawal symptoms, which typically occur 6–12 h after smoking cessation (21). Craving, withdrawal, mood, and anxiety were assessed before and after each patch administration and after each MRI scan.

### Imaging Analysis

#### MRI Data Acquisition

We analyzed existing data from two independent neuroimaging studies, so the MRI acquisition parameters were different for each cohort.

Cohort 1: Participants in Cohort 1 were scanned on a 3-T Siemens Trio scanner with a 32-channel head coil at McLean Imaging Center. Multi-planar rapidly acquired dual echo gradient-echo structural images used the following parameters: TR = 2.1 s, TE = 3.3 ms, slices = 128, matrix = 256 x 256, flip angle = 7°, resolution = 1.0 × 1.0 × 1.33 mm. The 6-min resting-state acquisition used the following parameters: 144 volumes, TR = 2.5 s, TE = 30 ms, slices = 42, flip angle = 90°, field of view = 448 × 448 mm^2^, and voxel size = 3.5 mm isotropic. Participants were asked to remain awake and to keep their eyes open for the duration of the scan.

Cohort 2: Participants in Cohort 2 were scanned on a 3-T Siemens Trio scanner with a 32-channel head coil at McLean Imaging Center. High resolution (1 × 1 × 1 mm^3^) T1-weighted MPRAGE images were acquired. During resting-state acquisition, participants were instructed to rest with eyes open for 6.2 min. Functional MR images were acquired with interleaved acquisition tilted −30° from the AC-PC line using a gradient-echo echoplanar imaging (EPI) sequence. Resting state data acquisition used the following parameters: 124 volumes, TR = 3 s, TE = 30 ms, slices = 47, flip angle = 85°, field of view = 504 × 504 mm^2^, and voxel size = 3.0 mm isotropic.

#### MRI Data Processing

All analyses were preprocessed using the DPABI toolbox (Data Processing and Analysis for Brain Imaging (22); http://rfmri.org/dpabi). As a quality control metric, data from any participant whose scans exceeded motion thresholds (3 mm translation or 3° rotation) were discarded. Individual time points with framewise displacement 0.5 mm were removed via scrubbing (23), and scans with 50% of volumes removed for framewise displacement were discarded. All data were preprocessed to remove motion (24-parameter), CSF signals, white matter signals, global signal, and overall linear trend. A bandpass filter was applied (0.01–0.08 Hz). Data were normalized using the DARTEL toolbox into Montreal Neurological Institute (MNI) space and smoothed with an 8-mm full-width half-maximum kernel. Analyses were conducted in a gray matter mask defined within the group.

#### Cohort 1: Network Discovery Using Multivariate Distance Matrix Regression

We conducted an assessment across all participants regardless of group (schizophrenia and controls) to identify shared and diagnosis-specific circuits of nicotine use. A multivariate pattern analysis of whole-connectome data (MDMR) was used to identify the strongest links between daily cigarette consumption and functional connectivity (17). This analysis occurs in two steps: the first step identifies any regions where daily cigarette consumption correlates with functional connectivity, and the second step (*post-hoc* testing) involves seed-based analysis of the identified region (see *ROI-Based Connectivity Analysis*) to determine the spatial pattern of connectivity it represents (17, 24, 25).

**Figure 1:**
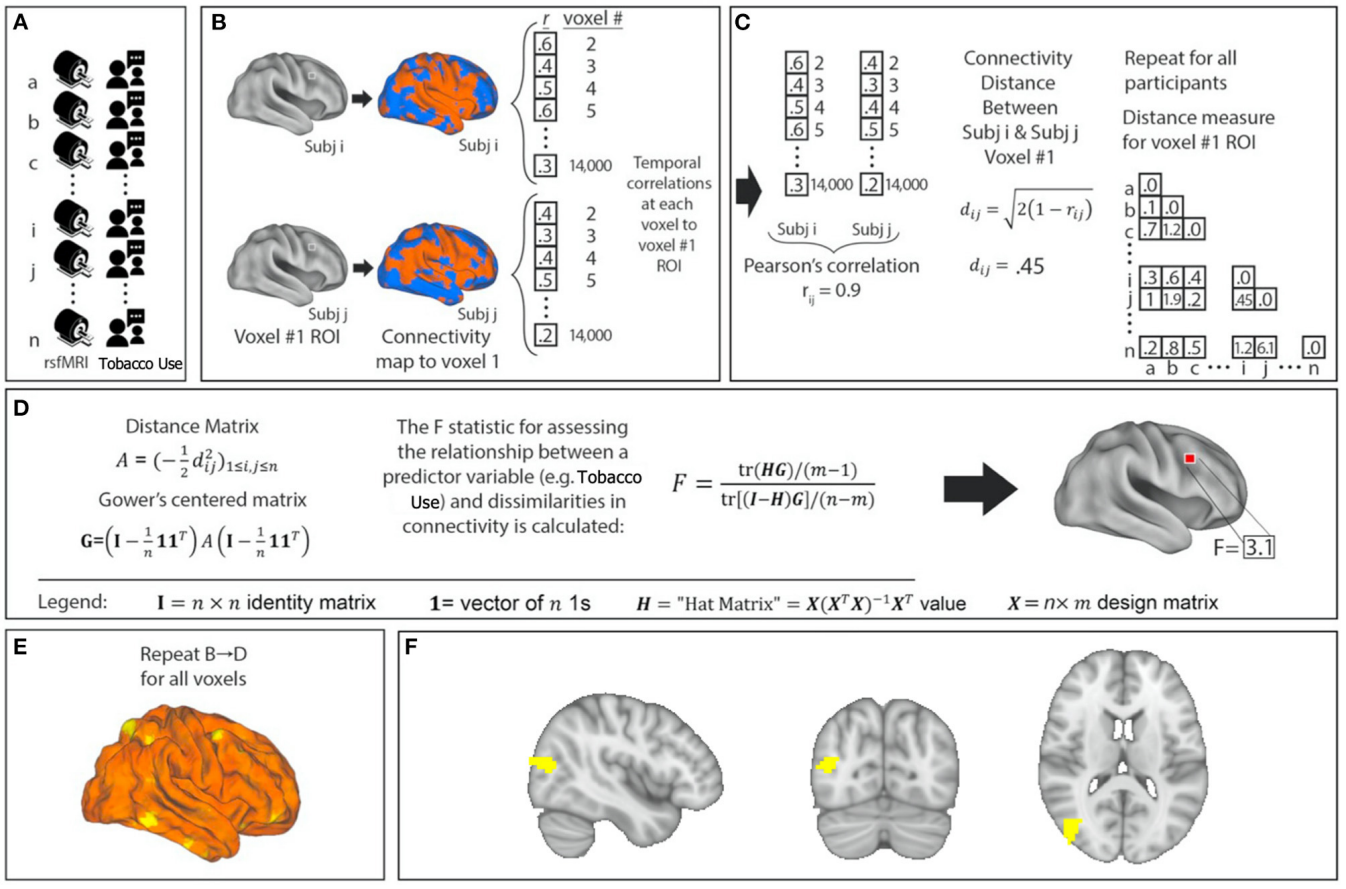
Network identification using Multivariate Distance Matrix Regression (MDMR). Cigarette consumption and resting-state functional MRI (rsfMRI) data were collected for each participant **(A)**. For each voxel in the brain, the voxel was used as a seed region to create a connectivity map for each participant **(B)**. These maps were compared with each other to create a subject-wise similarity matrix **(C)**. Daily cigarette consumption for each participant was then combined with the connectivity similarity matrix to produce a pseudo-F statistic, which characterizes how individual variation in cigarette consumption explains individual variation in functional connectivity **(D)**. This is repeated for all voxels **(E)**. Each MDMR voxel-wise result was then combined to produce a map of the ability of the connectivity pattern to predict a cigarette consumption score in each voxel **(F)**. A permutation test of the study subjects' labels can be used to test the significance of this pseudo-F statistic.

After preprocessing, resting-state fMRI data were analyzed with MDMR (Figure 1) (17). This method allows for an unbiased, data-driven approach to identifying phenotype-connectivity relationships. MDMR allows quantification of how a variable of interest (daily cigarette use here) is reflected in the distributed connectivity of individual voxels to the whole brain (i.e., at the finest resolution possible) without parcellating the brain into regions defined a priori (Figure 1). In brief, MDMR tests every voxel to determine if whole-brain connectivity to that voxel is more similar in individuals with similar values on an independent measure (daily cigarette consumption, cigarettes/day) than in individuals with dissimilar values. As described previously, MDMR occurs in several stages: First, scan and cigarette consumption measurements are collected from all participants (Figure 1A). Functional connectivity was calculated in the following way: a seed-to-voxel functional connectivity map is generated for each participant. These functional connectivity maps are correlated by calculating the temporal Pearson's correlation coefficients between each voxel, using its BOLD signal time-course, and all other gray matter voxels using a standardized gray matter mask included in the DPABI toolbox (Figure 1B). The temporal correlation coefficients for each voxel in the functional connectivity map are then correlated with the values of corresponding voxels in the maps generated for the other participants. This Pearson's correlation coefficient, *r*, is a measure addressing how similar the whole-brain functional connectivity to a specific voxel is, for each voxel, between patients. This value is used to calculate between-subject distance (or dissimilarity) using the metric *d*_*ij*_ = $\sqrt{2\left(1-r_{ij}\right)}$ where *i* and *j* are two subjects and *r* is the correlation coefficient above (Figure 1C). Third, we test the relationship between the independent variable of interest, here, daily cigarette consumption (cigarettes/day), and the inter-subject distances in connectivity generated at the previous stage. Broadly speaking, this process consists of an ANOVA-like hypothesis test between a variable of interest and a matrix of distances. This method was originally named multivariate distance matrix regression to study associations between gene expression and related variables. Shehzad et al. (17) adapted this method to test the relationship between variables of interest and a matrix of distances, the matrix being similarity between subject's whole-brain functional connectivity. This test first creates a distance matrix $A={\left(-\frac{1}{2}d_{ij}^{2}\right)}_{1\leq i,j\leq n}$ among n participants where d = the between-subject distance metric calculated above. Next, this matrix is used to create a Gower's centered matrix $G=\left(I-\frac{1}{n}11^{T}\right)A\left(I-\frac{1}{n}11^{T}\right)$, in which *n* is the number of participants, *I* is the *n* × *n* identity matrix, and 1 is a vector of *n* 1s. The *F* statistic for assessing the relationship between a predictor variable (e.g., daily cigarette consumption, cigarettes/day) and dissimilarities in connectivity is calculated as follows: For *m* predictor values, let ***X*** be a *n* × *m* design matrix of predictor values, and let *H* = *X*(*X*^*T*^*X*)^−1^*X*^*T*^ be the associated *n* × *m* “hat” matrix.

$F=\frac{tr\left(HG\right)/\left(m-1\right)}{tr\left[\left(I-H\right)G\right]/\left(n-m\right)}$ (Figure 1D). This process is repeated for every voxel. The result is a whole-brain map showing how significant the relationship between daily cigarette consumption measurements and functional connectivity is at every voxel (Figure 1E). From this generated map, ROIs for follow-up analysis are determined based on clusters of significant voxelwise *F*-statistics. To correct for multiple comparisons, a nonparametric permutation is calculated for voxels that exceed the significance threshold of *p* < 0.001 and clusters of such with an extent threshold of *p* < 0.05, with a null distribution calculated from 5,000 such permutations (17). This voxelwise threshold was selected to maximize the replicability potential. This approach has been used to examine the relationship between psychiatric pathology and connectivity (24, 25). We modeled the effect of daily cigarette consumption on functional connectivity while covarying for effects of chlorpromazine equivalents, age, and sex (Figure 1F). After the initial whole group analysis, we repeated this analysis for the schizophrenia group and the control group independently.

We conducted the MDMR analysis to identify anatomical regions where connectivity significantly varied with nicotine consumption. After identifying any MDMR regions, we then conducted follow-up seed-based connectivity (see *ROI-Based Connectivity Analysis*) analysis to examine the spatial distribution of these connectivity differences as in prior MDMR analyses (17, 26–28).

Importantly, MDMR identifies regions where connectivity correlates to a phenotype (e.g., daily cigarette consumption, cigarettes/day) but this initial analysis does not identify the direction of correlation or the spatial pattern. This process disregards spatial information about the voxels that gave rise to between-individual distances e.g., Two individuals may be very distant (dissimilar) in the functional connectivity of a single voxel in the parieto-occipital region, but is their dissimilarity driven by differences in parieto-occipital connectivity to the temporal lobes or medial prefrontal cortex or all of the above? The first step of MDMR does not display this information (17). In order to visualize this spatial information requires follow-on seed-based connectivity analysis. Shehzad et al. and others have termed this follow-on analysis “*post-hoc*” testing to make clear that this should not be considered independent hypothesis testing nor should it be considered independent validation of the original MDMR finding (17, 26–28).

#### ROI-Based Connectivity Analysis

To visualize spatial patterns of connectivity driving the results of MDMR, maps of connectivity to the region identified in MDMR were generated. This step identifies the spatial pattern of connectivity to the region identified in the MDMR analysis (17, 26–28). The time course of the BOLD signals from rsfMRI scans in the ROI identified in the MDMR process was extracted and whole brain connectivity maps were generated using DPABI. Using SPM12 (“SPM–Statistical Parametric Mapping,” http://www.fil.ion.ucl.ac.uk/spm) we regressed the z-transformed Pearson's correlation coefficient connectivity maps against daily cigarette consumption to generate spatial maps of how whole functional brain connectivity to the ROI varies with cigarette consumption. We measured ROI to ROI connectivity at this step by measuring BOLD correlation between an ROI defined by the MDMR-identified region and a DMN ROI (29). We then correlated connectivity between the MDMR-identified ROI and the identified brain networks with daily cigarette consumption.

#### Cohort 2: Network Effects of Acute Nicotine Administration

##### Whole-Network Connectivity Values

In Cohort 1, we observed a relationship between a pathologically expanded DMN topography and greater daily nicotine use. Based on this observation, we hypothesized that nicotine has a direct effect on the DMN. After considering which network features could be both modulated by nicotine and measured experimentally, we hypothesized that nicotine affects within-network functional connectivity. We therefore sought to test the ability of nicotine to acutely modulate DMN connectivity in an independent cohort. To test this hypothesis, we compared within-DMN mean functional connectivity at nicotine and placebo administration. We generated individual values of mean DMN functional connectivity by placing 6 mm spheres at coordinates that correspond to seven standard nodes of the DMN (posterior cingulate/precuneus, medial prefrontal, left lateral parietal, right lateral parietal, left inferior temporal, right inferior temporal, medial dorsal thalamus) [see (30) for coordinates]. The time course of the BOLD signals from the ROIs were correlated with each other and z-transformed to generate a 7 × 7 ROI to ROI connectivity matrix. A mean connectivity from this matrix was generated for each participant under each condition (nicotine or placebo). The output from this analysis was a mean connectivity value for the entire DMN for each participant at nicotine administration (FC_nicotine_) and at placebo administration (FC_placebo_). Change in whole-network functional connectivity (FC_nicotine_ – FC_placebo_) was calculated for each participant.

### Statistical Analyses

We correlated change in whole-network functional connectivity (FC_nicotine_ – FC_placebo_) with nicotine dose. We calculated Pearson correlation coefficients between measurements of functional connectivity and clinical variables. We used paired *t*-tests to compare change in connectivity between nicotine and placebo administration. We used a mixed effects repeated measures ANOVA to determine the effect of diagnosis, smoking status, and drug on changes in connectivity.

## Results

### Resting-State Network Organization Predicts Cigarette Consumption

**Figure 2:**
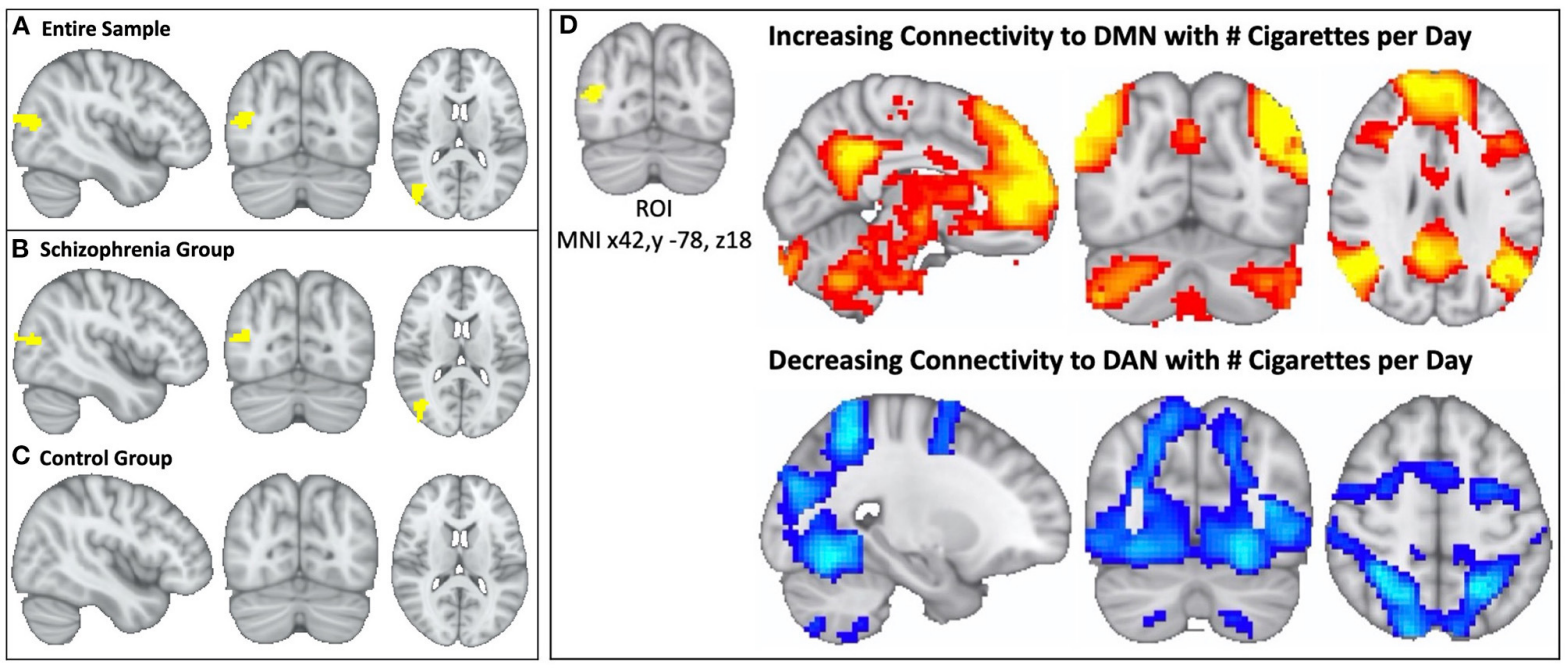
In schizophrenia, individual variation in DMN topography is linked to cigarette consumption: higher cigarette consumption is linked to both increased connectivity between parieto-occipital ROI and DMN and decreased connectivity between parieto-occipital ROI and the DAN. We identified a parieto-occipital region (Thresholded voxelwise *p* < 0.001, Cluster *k* = 77, MNI x42, y −78, z18, *p* < 0.05) where functional connectivity correlates with daily cigarette use in our combined sample of schizophrenia and control smokers **(A)**. In this region, increased functional connectivity correlates with greater daily cigarette consumption. We repeated this analysis in each diagnostic sub-group and observed a significant relationship between connectivity and cigarette use in the schizophrenia group (Cluster *k* = 52, *p* < 0.05) **(B)** but not in the neurotypical group **(C)**. After identifying the parieto-occipital region where functional connectivity correlated with daily cigarette use, we conducted follow-up seed-based connectivity analysis to examine the spatial pattern of how connectivity to this region correlates with nicotine consumption in the schizophrenia group. We observed that global functional connectivity to this region was positively correlated with the DMN and negatively correlated with the DAN **(D)**, which was readily apparent by visual inspection. Higher cigarette consumption was linked to both increased connectivity between the parieto-occipital ROI and DMN and decreased connectivity between the parieto-occipital ROI and the DAN.

Multivariate pattern analysis of the whole-connectome of Cohort 1 (18 schizophrenia, 17 controls) identified a single region (Cluster *k* = 77, centered at MNI × = 42, y = −78, z = 18) in the right parieto-occipital region where functional connectivity correlated with daily cigarette consumption (Figure 2A). When we repeated this analysis in each group (i.e., in schizophrenia alone and controls alone) we observed a significant relationship between connectivity and cigarette consumption in the schizophrenia group (*n* = 18, Cluster *k* = 52, Figure 2B) but no such relationship in the control group (*n* = 17, Figure 2C). This was observed despite the fact that groups were matched for severity of nicotine dependence. Further, there were no significant group-level differences in head motion (*p* = 0.99).

**Figure 3:**
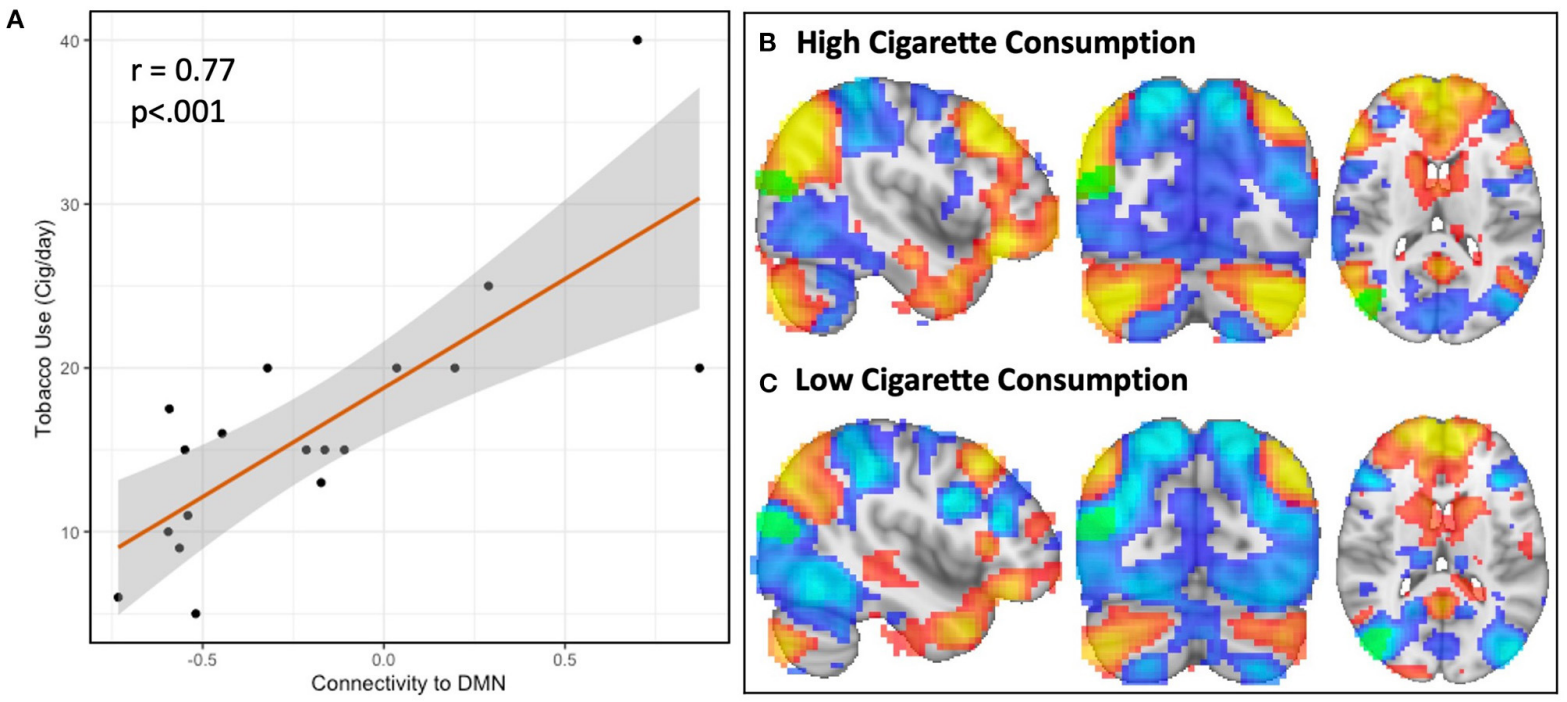
Resting-state network topography is strongly linked to cigarette consumption in schizophrenia. The spatial organization of resting-state networks is strongly linked to cigarette consumption in schizophrenia. In the first step of MDMR, we identified a parieto-occipital region where functional connectivity correlated with daily tobacco use (Figure 1). In order to determine the direction and spatial pattern of connectivity to this parieto-occipital region, we conducted follow-up seed-based analysis within the schizophrenia cohort. This region was connected to the default mode network (DMN), which was readily identified by visual inspection. We therefore measured the functional connectivity between our MDMR-identified region and the DMN. When we compared tobacco use (cigarettes per day) with connectivity to the DMN, we observed a linear relationship where greater daily cigarette consumption was significantly correlated with greater connectivity to the DMN [**(A)**, *r* = 0.77, *p* < 0.001]. This indicates that, with increasing daily cigarette consumption, the identified parieto-occipital region is increasingly connected to the DMN rather than to other networks. We determined this relationship was due to the topography of the DMN, as evidenced by two representative individuals **(B,C)**. Displayed here are the DMN (red) and dorsal attention network (DAN, blue) in two example individuals diagnosed with schizophrenia with either high **(B)** (40 cigarettes/day) and low **(C)** (5 cigarettes/day) cigarette consumption. In an individual with high daily cigarette consumption (3B), the DMN extends into the parieto-occipital region identified using MDMR (*k* = 52, MNI x = 42, y = −78, z = 18, green) while the DAN does not; in an individual with low daily cigarette consumption **(C)**, the DMN is external to this region and this region is part of the DAN. This link between network topography and cigarette consumption was not observed in a comparison group of nicotine-dependent controls.

In the follow-up analysis to determine the spatial pattern of how connectivity to the right parieto-occipital region correlates with nicotine consumption, we observed that functional connectivity to this identified region is positively correlated with the DMN and negatively correlated with the dorsal attention network (DAN) in the schizophrenia group (*n* = 18, Figures 2D, 3A). We observed a linear relationship where greater daily cigarette consumption was significantly correlated with greater connectivity to the DMN (Figure 3A, *r* = 0.77, *p* < 0.001). Notably, this linear relationship extended across both positive (correlation) and negative (anti-correlation) connectivity values. To elaborate: In some individuals activity in this MDMR identified region was correlated with the DMN (and anticorrelated with DAN) while in other individuals this region was correlated with DAN (and anticorrelated with DMN). This is consistent with spatial distribution (topography) of these networks co-varying with cigarette consumption in the schizophrenia group (*n* = 18, Figure 3). In an individual diagnosed with schizophrenia who smokes heavily, this parieto-occipital region was part of the DMN and not part of the DAN (Figure 3B). In a light smoking individual with schizophrenia, this relationship was reversed: the parieto-occipital region was instead part of the DAN and was external to the DMN (Figure 3C). In summary, with increasing daily cigarette consumption, this parieto-occipital region was increasingly likely to be a part of the DMN and less likely to be connected to the DAN, suggesting a difference in the *topography* of these two resting-state networks. Thus, we observed that individual-level differences in the spatial organization (i.e., topography) of the DMN and DAN in this region reflected individual variation in daily cigarette consumption in the schizophrenia group. In a large sample of healthy young adults, this parieto-occipital region is typically a part of the DAN, suggesting that the extension of the DMN into this region in heavy smokers with schizophrenia represents schizophrenia-specific pathologic organization (29).

To summarize, in schizophrenia, the strongest functional connectivity correlate of nicotine consumption in the brain is driven by variation in the topography of resting-state networks. In participants diagnosed with schizophrenia, individual variation in the spatial arrangement of these networks is linked to individual variation in nicotine consumption. Specifically, with greater daily cigarette consumption, we observed an expansion of the DMN into territory normally occupied by the DAN. This relationship between topography and nicotine use was not observed in neurotypical controls.

### The Relationship Between Acute Nicotine Administration and DMN Connectivity

In the first cohort we observed a strong relationship between cigarette use and the spatial organization of the DMN. This relationship was linearly related to the amount of daily nicotine use in participants diagnosed with schizophrenia such that greater daily cigarette use was associated with increased connectivity to the DMN (Figure 3A) and a pathologically expanded topography of the DMN (Figures 3B,C). However, the directionality of this association was unclear and had several different interpretations (see Discussion). We therefore sought to test if nicotine had a direct effect on DMN resting-state connectivity using an independent second dataset (Cohort 2).

Acute nicotine administration has been observed to decrease activity in the DMN during a task (31) and at rest (32). Therefore, we hypothesized that (1) nicotine would acutely affect the temporal correlation of spontaneous activity (i.e., functional connectivity) of the DMN in a dose-dependent manner and (2) this effect would preferentially affect participants with schizophrenia. In Cohort 2, 24 participants (12 SZ, 12 HC) were enrolled in a randomized, placebo-controlled crossover study of transdermal nicotine patch (Table 2). Participants underwent resting-state fMRI and clinical characterization at each timepoint as described in Materials and Methods.

**Figure 4:**
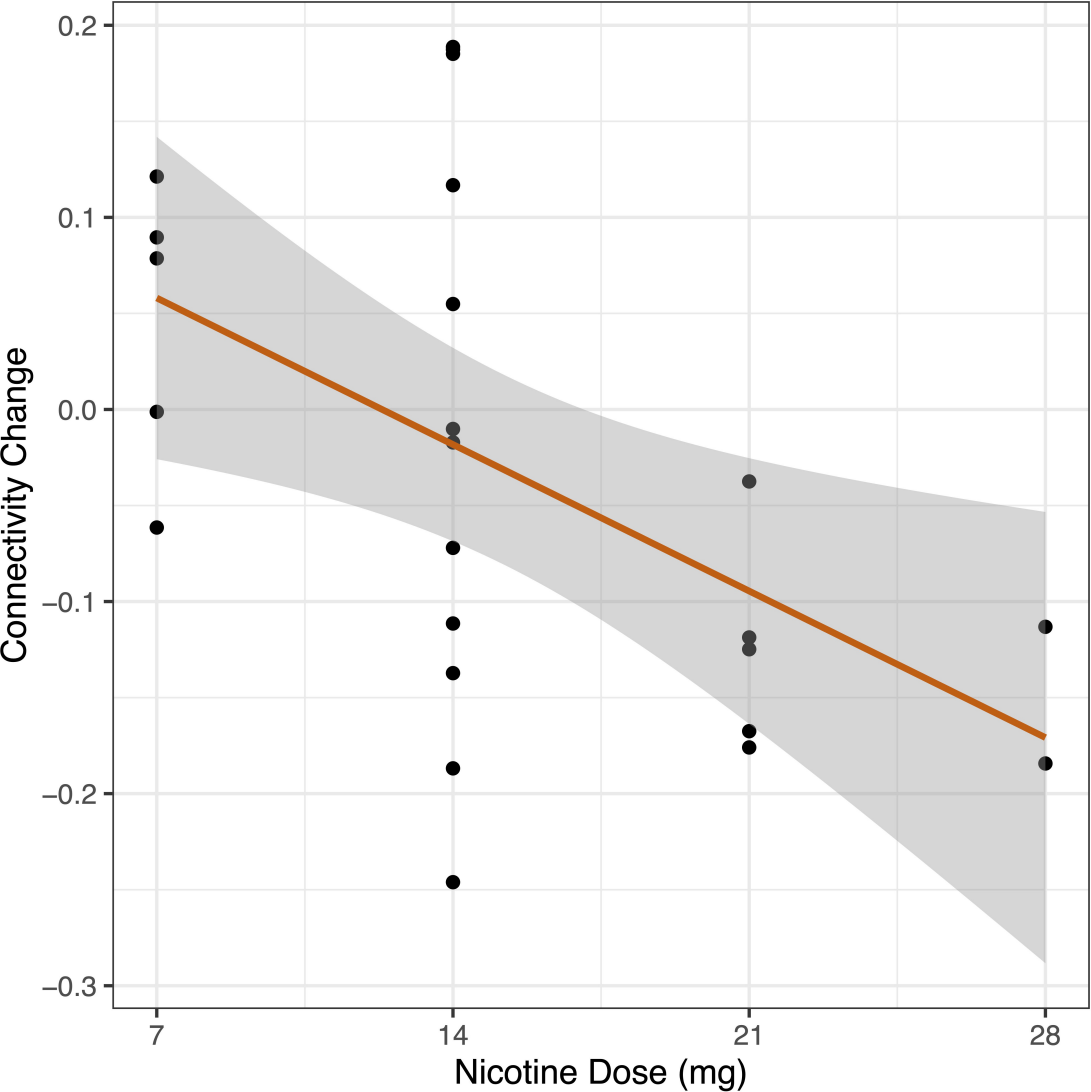
Dose-response relationship between nicotine and DMN connectivity change. In a cohort of schizophrenia and control participants, we measured whole DMN functional connectivity twice: once in the placebo condition and once with nicotine patch. Change in whole DMN functional connectivity was calculated between these two administrations (FC_nicotine_ – FC_placebo_). Dosage of nicotine patch varied between individuals based on smoking status (nicotine-dependent, *n* = 8, range 14–28 mg; non-nicotine dependent, *n* = 16, range 7–14 mg). We correlated DMN network connectivity change to administered nicotine dose across the combined sample (*n* = 24). We observed a linear relationship between increased nicotine dose and greater reduction in DMN connectivity across the entire sample (*r* = −0.50, *p* = 0.012).

After generating an entire DMN-network measurement of functional connectivity using seven ROIs defined as part of the DMN, we measured whole DMN functional connectivity change in Cohort 2 in relation to nicotine dose across the combined sample (*n* = 24). We observed a linear relationship between higher nicotine dose and greater reduction in DMN connectivity (*R* = −0.50, 95% CI −0.75 to −0.12, *p* = 0.012) (Figure 4).

### Sensitivity Analyses

Given our mixed sample of nicotine-dependent (*n* = 8) and non-nicotine dependent (*n* = 16) individuals in Cohort 2 and the potential confounding effect of nicotine withdrawal symptoms, we performed correlations between ratings of nicotine withdrawal among nicotine-dependent participants and DMN connectivity. DMN connectivity did not correlate with post-scan nicotine withdrawal at the placebo (*R* = 0.37, 95% CI −0.53 to 0.88, *p* = 0.42) or nicotine timepoint (*R* = −0.11, 95% CI −0.76 to 0.64, *p* = 0.80). There was no correlation between change in DMN connectivity and change in post-scan withdrawal symptoms (*R* = 0.17, 95% CI −0.67 to 0.82, *p* = 0.72). When we controlled for post-scan withdrawal symptoms between change in DMN connectivity and nicotine dose, there was no significant partial correlation (*r* = −0.17, test statistic = −0.35, *p* = 0.75). There was a main effect of drug on head motion, such that there was less head motion with nicotine administration (*p* = 0.02). However, there were no significant differences in head motion between participants with schizophrenia and controls at nicotine (*p* = 0.49) or placebo (*p* = 0.84) conditions, nor were there any no group x drug effects on head motion (*p* = 0.61). Further, there was no effect of smoking status on head motion (*p* = 0.34). In summary, our findings do not appear to be driven by potential withdrawal symptoms during the placebo condition. This is consistent with the study protocol, which was designed to avoid peak withdrawal symptoms. Indeed, reported withdrawal was minimal, and there was no difference between median post-scan withdrawal scores at nicotine (2/24) or placebo administration (2/24).

After observing significant effects of nicotine on global DMN connectivity in Cohort 2, we interrogated connectivity between individual nodes of the DMN to determine if the network-wide effect was driven by region-specific alterations in connectivity. We did not identify any specific nodes in the DMN that explained this result (Table 3). Nicotine dose was correlated with a generalized decrease in connectivity across the entire DMN. There was a significant correlation between change in DMN connectivity and nicotine dose between seeds in the precuneus/PCC and bilateral temporal lobes (*p* < 0.05), but this does not survive correction for multiple comparisons.

**Table 3:** Node-specific correlation values of DMN connectivity change and nicotine dose.

	**Precun/PCC**	**mPrefrontal**	**Lparietal**	**Rparietal**	**Ltemporal**	**Rtemporal**	**mdThalamus**
Precun/PCC	−	−0.19	−0.20	−0.20	−0.41^*^	−0.48^*^	−0.17
mPrefrontal	−0.19	−	−0.13	−0.27	−0.21	−0.39	−0.17
Lparietal	−0.20	−0.13	−	−0.26	−0.38	−0.38	−0.07
Rparietal	−0.20	−0.27	−0.26	−	−0.23	−0.23	−0.14
Ltemporal	−0.41^*^	−0.21	−0.38	−0.23	−	−0.14	0.24
Rtemporal	−0.48^*^	−0.39	−0.38	−0.23	−0.14	−	−0.29
mdThalamus	−0.17	−0.17	−0.07	−0.14	0.24	−0.29	−

*Precun/PCC, precuneus and posterior cingulate cortex; mPrefrontal, medial prefrontal; Lparietal, left parietal; Rparietal, right parietal; Ltemporal, left temporal; Rtemporal, right temporal; mdThalamus, medial dorsal thalamus. Pearson correlation coefficients were calculated and Z-transformed*.

* *p < 0.05 uncorrected*.

### Diagnosis-Specific Effects of Nicotine on Default Mode Network Connectivity

We then considered if the effect of nicotine on DMN connectivity in Cohort 2 was more pronounced in schizophrenia (*n* = 12) compared to controls (*n* = 12).

**Figure 5:**
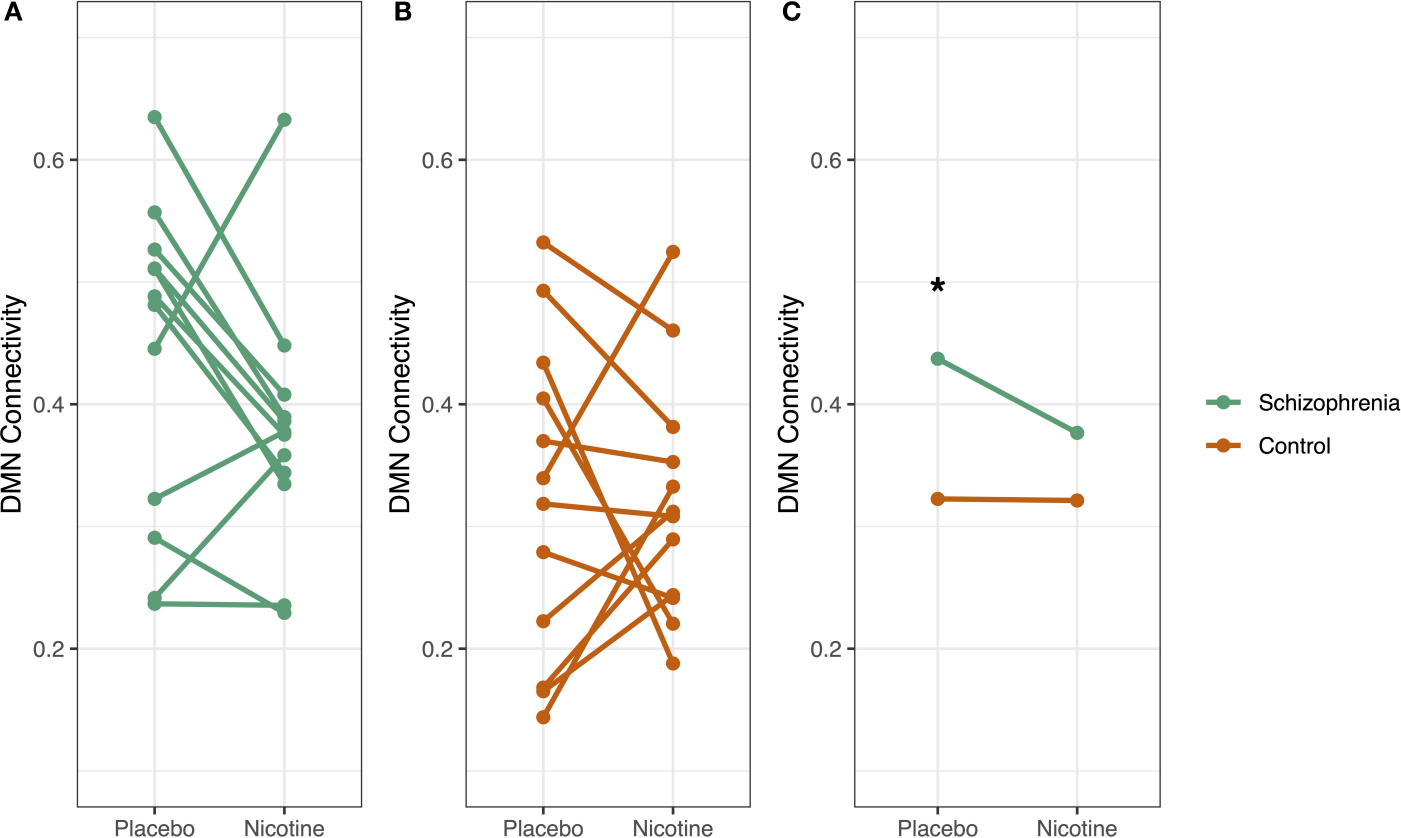
Diagnosis-specific changes in DMN connectivity by drug condition. Whole default mode network (DMN) connectivity for each participant was measured at two timepoints: at placebo and at nicotine administration. In schizophrenia **(A)**, DMN connectivity decreased with nicotine administration, but this pattern was not evident in controls **(B)**. Subjects with schizophrenia had significantly higher DMN connectivity compared to controls in the placebo condition (*p < 0.05) but not with nicotine administration **(C)**. There was a trend-level interaction between diagnosis and smoking on DMN connectivity (*p* = 0.09), where smoking and schizophrenia diagnosis both increased DMN connectivity.

During the placebo condition in Cohort 2, there was a significant effect of diagnosis on mean whole-DMN connectivity. We observed mean whole-DMN hyperconnectivity in schizophrenia compared to controls (0.44 vs. 0.32, *p* < 0.05), consistent with prior findings in schizophrenia (33). However, during nicotine administration, DMN connectivity was no longer significantly different between groups (0.37 vs. 0.32, *p* = 0.19) (Figure 5).

We observed evidence that nicotine dose had a significant effect on DMN connectivity which was more pronounced in schizophrenia in Cohort 2. A mixed-effects repeated measures ANOVA was performed to assess the interaction of diagnosis, smoking status, and drug (nicotine vs. placebo) on DMN connectivity. The schizophrenia group had significantly higher DMN connectivity (*p* < 0.05) than controls. There was a trend-level interaction between diagnosis and smoking on DMN connectivity (*p* = 0.09), where smoking and schizophrenia diagnosis both increased DMN connectivity. There was no significant effect of drug order.

## Discussion

We identified a novel brain basis for the high prevalence of nicotine dependence in schizophrenia. We observed that variation in nicotine consumption is linked to the spatial organization of two resting-state networks, the DMN and the DAN in a right parieto-occipital region. Specifically, higher amounts of daily tobacco consumption were linked to expansion of the DMN into territory normally occupied by DAN in healthy controls (29). This relationship between tobacco consumption and topography was observed only in participants with schizophrenia. Individual differences in network topography have been previously observed (18, 34–39). Only recently have these topographic variations been linked to behavioral/cognitive phenotypes (24, 25, 40). Our report of a link between network topography and substance use in schizophrenia is novel.

There are several possible interpretations for the association between DMN/DAN network organization and tobacco consumption. One explanation is that tobacco use changes brain network spatial organization. This is unlikely, as such an explanation would presumably be observed in both neurotypical nicotine using individuals and in those with schizophrenia, which was not observed in the current study. Given the link between nicotine use and network topography was found only in those with schizophrenia, it is more likely that the pathophysiology of schizophrenia changes the organization of the DMN. The observable result of this is (1) previously described DMN within-network hyperconnectivity in schizophrenia and (2) changes in network spatial organization observed in our sample. Nicotine may correct DMN within-network hyperconnectivity such that individuals with schizophrenia are drawn to smoking and therefore use in doses that are commensurate with the degree of network disruption.

If the degree of network pathology leads to cigarette smoking proportional to underlying pathology, then acute nicotine would be expected to (1) have a direct effect on the network and (2) demonstrate a dose-response relationship between nicotine dose and network connectivity. In order to differentiate these possible explanations, we investigated the effects of acute nicotine administration on DMN connectivity.

We examined the effect of acute nicotine administration on the DMN and observed a dose-response relationship between nicotine dose and DMN connectivity (Figure 4). Although it has been previously observed that acute nicotine administration decreases DMN activity in nicotine-dependent individuals (31) and decreases DMN connectivity in non-smoking controls (32), a dose-response curve has not been reported. The nicotine-induced reduction in DMN connectivity occurred across the entire network rather than being driven by an individual node of the DMN. We then examined differences in DMN connectivity by diagnosis. Participants with schizophrenia demonstrated DMN hyperconnectivity, a finding consistent with existing literature (33). This diagnosis-specific increased DMN connectivity was no longer significant after acute nicotine administration, consistent with a theory in which DMN network pathology is an underling cause of nicotine use in schizophrenia. We observed that nicotine administration was associated with a greater reduction of DMN connectivity in the schizophrenia group than the control group, a trend-level drug by diagnosis interaction (p = 0.09). Our observation in Cohort 2 that nicotine normalizes DMN hyperconnectivity in schizophrenia was confirmed when we subsequently analyzed DMN connectivity in the initial cohort. We observed that in Cohort 1, there was no difference in DMN connectivity between smokers with schizophrenia and control smokers, suggesting that nicotine may be normalizing connectivity to control levels.

Our results imply a key role of the DMN in nicotine dependence in schizophrenia. Others have previously identified the involvement of the DMN in nicotine dependence in individuals without a co-morbid psychiatric disorder (31, 32, 41–43). However, we observed diagnosis-specific effects of nicotine on the DMN in schizophrenia that were not observed in controls. Notably absent from our data-driven analysis was any significant link between tobacco consumption and salience/reward network connectivity.

**Figure 6:**
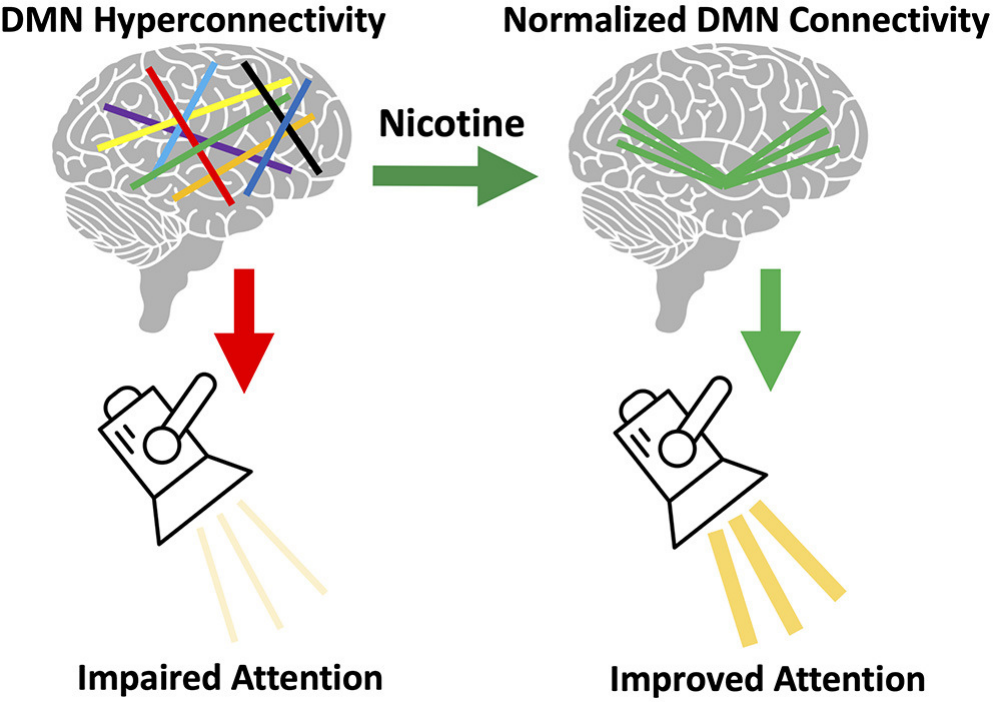
Proposed model of brain circuitry driving nicotine use in schizophrenia. We observed default mode network (DMN) hyperconnectivity in our sample of individuals with schizophrenia, consistent with prior literature (33). We also observed that acute nicotine administration normalized DMN hyperconnectivity in schizophrenia. Based on this data, we hypothesize that individual variation in the magnitude of this DMN disruption gives rise to: (a) variation in the severity of network disorganization and (b) variation in severity of nicotine use. We propose that the DMN mediates the relationship between nicotine use and cognition in schizophrenia. DMN hyperconnectivity is linked to poor attentional performance (45) and, in healthy controls, nicotine can both reduce DMN connectivity and improve attention (11). We propose that DMN connectivity mediates the relationship between nicotine use and attentional performance and that disruption of this network in schizophrenia drives the need for nicotine to correct network pathology (and cognition).

However, questions remain: is there a cognitive or behavioral benefit from nicotine's normalization of DMN connectivity? Although our current data is unable to answer this question directly, we hypothesize there is a cognitive benefit from normalized DMN connectivity. Cognitive deficits have been correlated with increased DMN connectivity (44). Nicotine improves attention in schizophrenia (11). Attentional performance is directly related to the ratio of DAN connectivity to DMN connectivity, such that higher DMN connectivity corresponds with worse performance (45). We therefore hypothesize that nicotine use *in schizophrenia* is driven by a need to normalize DMN connectivity and thereby correct attentional deficits (Figure 6). We are unable to test this hypothesis using the current data, as no cognitive measures were collected. Another unanswered question is how this altered DMN connectivity occurs. This may occur during development, but additional research studying network connectivity in the prodrome may provide further insights.

Our study has several strengths, notably that we used an agnostic, data-driven approach that was successful in identifying schizophrenia-specific effects. Existing studies of nicotine and schizophrenia have not explained the diagnosis-specific relationships between nicotine and biology. This is the first study to examine individual variation in nicotine use to identify a biological correlate for nicotine dependence in schizophrenia. Our analysis was strengthened by the use of individual variation in nicotine use and continuous variables (cigarettes per day, nicotine dose) rather than categorical comparisons between smokers and non-smokers.

This study uniquely allowed us to probe issues of causality by use of a nicotine intervention in Cohort 2. If two variables are causally linked experimentally, then manipulating one variable should induce change in the other. In Cohort 2, we experimentally manipulated administered nicotine dose and measured network connectivity. We observed that nicotine directly acted upon the DMN, a network which our first analysis (Cohort 1) linked to nicotine dependence. This allowed us to identify the direction of the relationship between nicotine and DMN hyperconnectivity: that nicotine reduced—and actually normalized—DMN hyperconnectivity in the schizophrenia group. A key strength of this approach was the use of an independent second cohort of a nicotine intervention and measures of within-subjects change which corroborated the results of our data-driven analysis in the first cohort. This complementary combination of studies is rare and powerful, despite the small sample of each cohort individually.

The major limitation of this study is the small sample size, which increases the risk of type I and type II errors. Although the crossover study design in Cohort 2 allows for detection of within-subject drug effects, Cohort 2 was not powered to observe a significant drug x diagnosis interaction. We show significant group-level differences of the DMN with placebo that are no longer significant after nicotine administration. This lack of a difference is attributable to the change in connectivity observed in the schizophrenia population (Figure 5), but there are not enough individuals in the group to achieve statistical significance for a drug x diagnosis interaction. Given the heterogeneity of schizophrenia, is also possible that these results may only apply to a subpopulation of people with schizophrenia. Our preliminary results require replication in a larger cohort.

There are several other limitations to our study, including the difference in age between controls and participants with schizophrenia in Cohort 1, although the multivariate pattern analysis controlled for age. Another limitation of the study is the relatively short resting scan sequence used in both cohorts (6.2 min, TR = 3 s). Another limitation of this study is the lack of cognitive measures, which would be important to further elucidate the consequences of nicotine's effects on DMN connectivity.

Our analysis was not powered to detect differences between nicotine-dependent and non-nicotine-dependent populations. The schizophrenia and control groups were imperfectly matched, leading to a disproportionate percentage of nicotine dependent individuals in the schizophrenia group, which could bias the results such that they were experiencing nicotine withdrawal during the placebo condition. However, in a control analysis, we did not observe any significant effects of withdrawal on connectivity, and overall there were low levels of reported withdrawal. While DMN connectivity would be expected to correlate with levels of withdrawal (41), we did not observe this relationship, likely due to the fact that our study was designed to avoid peak withdrawal symptoms, and as a result, participants experienced minimal withdrawal symptoms. It is also possible that we did not observe a correlation between DMN connectivity and withdrawal due to the small sample size. Further, because the administered nicotine dose was related to smoking severity, we are unable to completely isolate an effect of nicotine administration that is independent of severity of nicotine dependence. Group differences could have been influenced by antipsychotic medication. In the future, we call on the large-scale data community to begin to include nicotine use data to enhance the reproducibility of these findings.

The current work identified DMN hyperconnectivity in the schizophrenia group, which was normalized by acute nicotine administration, thus indicating a direct, schizophrenia-specific effect of nicotine on network connectivity. However, the precise cognitive or behavioral consequence of nicotine-induced changes in mitigating DMN hyperconnectivity has not been elucidated. Future studies should relate changes in attentional performance to change in DAN/DMN connectivity pre- and post-nicotine. If network hyperconnectivity is corrected by nicotine, which, in turn, improves cognitive deficits, correcting this network problem may improve cognition. Neuromodulation, such as repetitive transcranial magnetic stimulation, could be one way to correct this network hyperconnectivity and provide a potential treatment for nicotine dependence in this population.

## Data Availability Statement

The raw data supporting the conclusions of this article will be made available by the authors, without undue reservation.

## Ethics Statement

The studies involving human participants were reviewed and approved by Massachusetts General Hospital and McLean Hospital Institutional Review Boards. The patients/participants provided their written informed consent to participate in this study.

## Author Contributions

RB, MH, AJ, LM, and HW contributed to conception and design of the study. AB, RB, UN, and HW performed the neuroimaging analysis. HW performed the statistical analysis and wrote the first draft of the manuscript. All authors contributed to manuscript revision, read, and approved the submitted version.

## Funding

This work was supported by NIMH R01MH116170 (RB); NIMH R01MH111868 and NIMH R01MH117063 (MH); NIDA 1K02DA042987 and NIDA K01DA029645 (AJ); NIMH K23MH110564, NARSAD Young Investigator Award, Brain and Behavior Research Foundation, Pope-Hintz Fellowship Award, McLean Hospital, Dupont-Warren Fellowship Award, and Harvard Medical School (LM); and the Sidney R. Baer, Jr. Foundation, and the Norman E. Zinberg Fellowship in Addiction Psychiatry Research, Harvard Medical School (HW).

## Conflict of Interest

AJ is a consultant for Axial Biotherapeutics for activities unrelated to this work. The remaining authors declare that the research was conducted in the absence of any commercial or financial relationships that could be construed as a potential conflict of interest.

## Publisher's Note

All claims expressed in this article are solely those of the authors and do not necessarily represent those of their affiliated organizations, or those of the publisher, the editors and the reviewers. Any product that may be evaluated in this article, or claim that may be made by its manufacturer, is not guaranteed or endorsed by the publisher.
